# A Hybrid Virtual Fracture Clinic is Safe and Efficacious in the COVID-19 Era: Stay at Home and Save Lives

**DOI:** 10.7759/cureus.14849

**Published:** 2021-05-05

**Authors:** Anirudh Sharma, Muhammad I Butt, Bisola Ajayi, Simon Perkins, Shamim Umarji, Caroline Hing, Darren F Lui

**Affiliations:** 1 Trauma and Orthopaedics, St. George's University Hospitals NHS Foundation Trust, London, GBR

**Keywords:** fracture clinic, virtual clinic, hybrid clinics, covid-19, remote consultations

## Abstract

Introduction

The coronavirus disease 2019 (COVID-19) pandemic necessitated a change in the manner outpatient fracture clinics are conducted due to the need to reduce footfall in hospitals. While studies regarding virtual fracture clinics have shown these to be useful and effective, they focus exclusively on remote consultations. However, our service was bespoke to the patient - either a face-to-face, a telephone consultation or both, depending on patient need - a ‘hybrid virtual fracture clinic' (HVFC). We report patient satisfaction and outcomes with this service from the first wave of the pandemic.

Methods

We retrospectively interviewed patients who availed of the HVFC service at our institution during the first two weeks of national lockdown in England from March 23 to April 5, 2020. The number and type of consultations, patient vulnerability to COVID-19, and type of management (surgical vs non-surgical) were among the factors taken into consideration. Patient experience was assessed using the Net Promoter Score (NPS), Customer Effort Score (CES), and Customer Satisfaction Score (CSS) on a scale of 0-10. Patient-reported outcomes were assessed using the EuroQol-5D-5L score (including EQ Visual Analogue Scale {EQ-VAS} scoring on a scale of 0-100).

Results

The mean overall NPS, CES, and CSS for the service were 7.32, 7.24, and 7.49, respectively. The mean self-reported EQ-VAS rating was 77.5. Of 442 consultations, 246 were conducted virtually; 10% were face-to-face, 29% virtual, and 61% were hybrid consultations.

The HVFC resulted in a 55.65% reduction in footfall. Statistical analysis showed no significant difference across any outcome measure when compared between hybrid, virtual, and face-to-face consultations. Patients vulnerable to COVID-19 and those who did not require surgery tended to report better overall scores.

Conclusion

Our study indicates that the HVFC format can reduce patient footfall significantly (>50%) while providing effective and satisfactory outpatient care. There appears to be no difference in patient-reported outcomes between face-to-face consultations and hybrid or virtual consultations. Patients would recommend HVFC to family and friends, found it was easy to use, and reported good satisfaction with the service.

## Introduction

In March 2020, the World Health Organization (WHO) declared the coronavirus disease 2019 (COVID-19) viral infection as a pandemic caused by the severe Acute respiratory syndrome-coronavirus 2 (SARS-CoV2), belonging to the coronaviridae family [1,2]. Since then, several countries including the United Kingdom have suffered two or more waves of the pandemic, with many regions across the world still grappling with a surge in infections. Early reviews confirmed the virus to be highly transmissible with a mortality rate between 1.4% and 2% [3]. In the United Kingdom, social distancing guidelines, lockdowns, and standard operating procedures were outlined by the government and other organisations as infection rates and hospital admissions of COVID-19 patients soared during March-April 2020. The UK National Health Service (NHS) came under increasing pressure, and the government issued the public message during the lockdown to ‘Stay at Home. Protect the NHS. Save Lives.’ The British Orthopaedic Association (BOA) issued clinical guidelines for patient management using remote consultations [4]. These included delivering follow-up appointments by telephone or video call, and the non-operative treatment of fractures where possible, particularly for upper limb fractures. NHS guidelines also specified patients who would not require physical examination or tests, after carrying out the risk assessment and excluding exception criteria [5].

Keeping in line with these guidelines, virtual clinics were initiated and/or expanded in various hospitals in the United Kingdom. Telephone consultations and virtual clinics were previously under practice and have been reviewed in various studies [6,7]. The requirement to compare the clinical and cost-effectiveness of virtual clinics to face-to-face clinics was emphasised by the National Institute for Health and Care Excellence (NICE) in 2016 [7].

The importance of virtual clinics in delivering healthcare to remote locations is known. While previous studies regarding virtual fracture clinics have shown these to be useful, effective, and more economical, they focus exclusively on remote consultations [6,7]. However, the present pandemic necessitated the use of virtual clinics with the main aim of reducing patient footfall in hospitals.

The Trauma and Orthopaedics department at our institution started a 'hybrid virtual fracture clinic' (HVFC) service that was bespoke to the patient - either a face-to-face or telephone consultation, or both, depending on the patient requirement. Patients were informed regarding the mode of consultation appropriate for their need, depending on their vulnerability to the coronavirus, orthopaedic condition, and any particular requirements for management of the same. In the present article, we review this HVFC model using objective patient satisfaction scores and patient-reported outcomes for this novel service.

## Materials and methods

This was a retrospective study conducted at the outpatient department of a Level 1 Major Trauma Centre (MTC) in London. Patients who were booked to attend the fracture clinic service during the first two weeks of the national lockdown in the United Kingdom (March 23, 2020, to April 5, 2020) during the coronavirus pandemic were included in the analysis. Patients were contacted and the study questionnaire was conducted by telephone by the authors (AS and MIB) between June and July 2020. Verbal consent was obtained from all patients prior to the questionnaire. The study was registered with the institution's clinical audit department. Two follow-up phone-call attempts were made for unavailable patients. 

All patients over the age of 18 years were included in the study. Those less than 18 years of age and those who did not attend (DNA) the appointment/were not contactable at the time of appointment, were excluded. In elderly patients or those with dementia, responses from their primary carer were accepted. We noted the total number of virtual (telephone) and/or face-to-face consultations each patient attended up until the time of the questionnaire and used this data to later differentiate patients who had only virtual, only face-to-face, or ‘hybrid’ consultations. We also noted the type of condition (upper limb, lower limb, or spine/pelvis/other), whether these patients were discharged from the HVFC or still under follow-up, and whether they required surgery as part of their treatment. We noted the number of new patients, and how many of these had their first appointment within 72 hours of their injury. Patients were classified as vulnerable or non-vulnerable to COVID-19 based on the WHO criteria, taking into account their age and medical co-morbidities. These criteria have also been published and followed by the NHS [5]. 

Patient experience of the HVFC was assessed using a validated questionnaire, which included the Net Promoter Score (NPS), Customer Effort Score (CES), and Customer Satisfaction Score (CSS). These scores were evaluated on a scale of 1-10, with 1 being the worst and 10 being the best possible score. The NPS asks patients to rate their likelihood to recommend a service to a friend or colleague [8]. The CES is a single item metric that measures how much effort a customer/patient has to exert to get an issue resolved, a request fulfilled, a service acquired or a question answered [9]. The CSS is a measurement of overall customer/patient satisfaction with a certain service or product [10].

Moreover, patients were also asked about the likelihood of their using the HVFC format in the absence of a pandemic, and score this on a scale of 1-10, with 1 being not likely at all, and 10 being extremely likely. 

Patient-reported outcomes were assessed using the validated EuroQol Group 5D-5L (EQ-5D-5L) Score, consisting of the EQ-5D descriptive system and the EQ Visual Analogue Scale (EQ-VAS). The descriptive part includes questions about the patients’ ease of mobility, self-care, routine activities, pain/discomfort, and anxiety or depression. The EQ-VAS requires patients to score their own health on a scale of 1-100, with 1 being the worst and 100 being the best possible health [11].

The anonymity of all the recorded data was ensured during its processing and electronic analysis. Data entry was done in a Microsoft Excel spreadsheet and the final analysis was done using Statistical Package for Social Sciences (SPSS) software (IBM SPSS Statistics for Windows, Version 21.0. Armonk, NY: IBM Corp). The comparison of the net promoter score, customer effort score, customer satisfaction score, and self-rated EQ-VAS scores between different modes of consultations was done using the Kruskal Wallis test. Comparison of the descriptive components of the EQ-5D-5L (which were qualitative parameters) was analysed using the Fisher’s exact test. A p-value of less than 0.05 was considered significant.

## Results

Of 269 possible adult age group patients that attended the HVFC during the study period, 171 patients responded to the telephone call and the questionnaire. Of these, 70 (40.9%) were males while 101 (59.1%) were females. The mean age was 46.49 years (range 18-93 years). 

Table 1 shows the distribution of patients based on vulnerability to COVID-19, surgical or non-surgical management, and follow-up status at the time of the questionnaire.

**Table 1:** Distribution of HVFC patients in vulnerable, surgical, and discharged categories HVFC: hybrid virtual fracture clinic

N = 171	Yes	No
Vulnerable?	44 (25.7%)	127 (74.3%)
Surgical management?	53 (30.9%)	118 (69.1%)
Discharged from HVFC?	112 (65.5%)	59 (34.5%)

**Figure 1:**
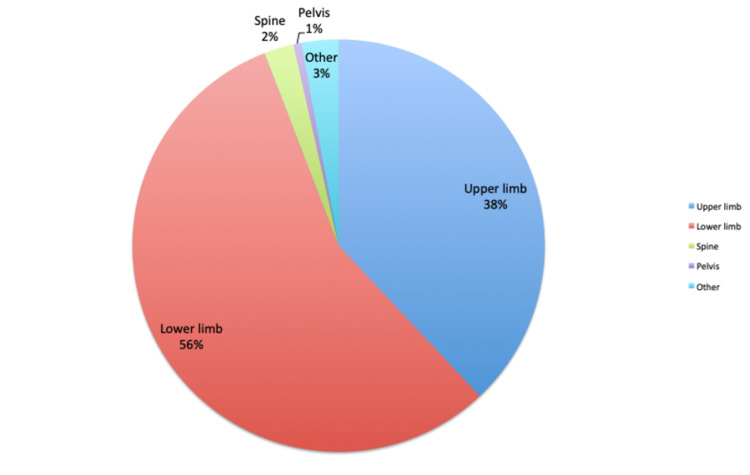
Distribution of orthopaedic conditions in the HVFC HVFC: hybrid virtual fracture clinic

Patients with lower limb conditions constituted the majority of those in HVFC (Figure 1).

The mean NPS, CES, and CSS of the hybrid virtual fracture clinic scored on a scale of 1-10 were 7.32, 7.23, and 7.49, respectively. The mean likelihood of using the HVFC in the absence of a pandemic was 6.46 (Table 2).

**Table 2:** The mean, median, and interquartile range of the NPS, CES, CSS, and likelihood of using the HVFC format in the absence of a pandemic, scored on a scale of 1-10 COVID: coronavirus disease; HVFC: hybrid virtual fracture clinic

	Net promoter score	Customer effort score	Customer satisfaction score	Non-COVID likelihood
Mean	7.32	7.23	7.49	6.46
Median	8	7	8	7
Interquartile range (Q3-Q1)	2	3	3	3

**Figure 2:**
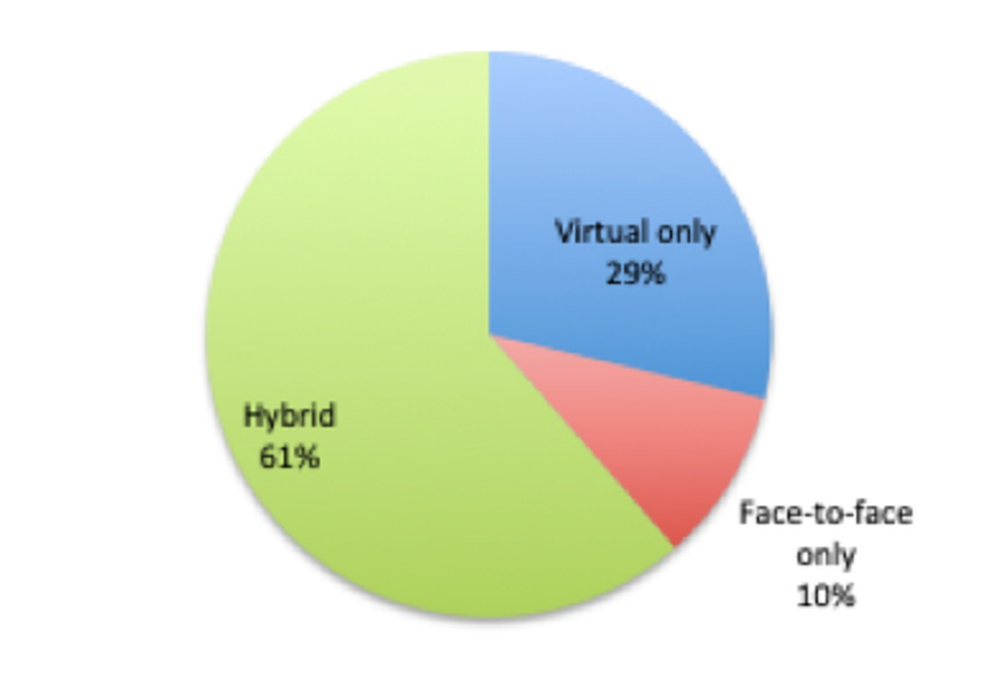
Distribution of HVFC patients based on the mode of consultation HVFC: hybrid virtual fracture clinic

There were 109 new patients referred from the accident and emergency department, of which 56 (51.3%) had their first consultation within 72 hours. There were 442 consultations, of which 246 were conducted virtually, saving 246 (55.65%) hospital visits. Overall, 17 patients (10%) had only face-to-face consultations, 49 (29%) had only virtual consultations, while 105 patients (61%) had hybrid consultations (Figure 2).

A Kruskal Wallis test was done to compare the outcomes of different modes of consultation (face-to-face, hybrid, and virtual). There was no statistically significant difference across NPS, CES, CSS, or EQ-VAS ratings between the groups (Table 3).

 

**Table 3:** Comparison of outcome measures between types of consultations using a Kruskal Wallis test NPS: Net Promoter Score; CES: Customer Effort Score; CSS: Customer Satisfaction Score; EQ-VAS: EuroQol Visual Analogue Scale

Parameter	Face to face only (n=17)	Virtual only (n=49)	Hybrid consultation (n=105)	Total	P-value
NPS (Mean ± SD)	7.53 ± 1.91	7.29 ± 1.77	7.3 ± 1.86	7.32 ± 1.83	0.88
CES (Mean ± SD)	7.12 ± 2.03	7.2 ± 2.11	7.28 ± 1.86	7.24 ± 1.94	0.94
CSS (Mean ± SD)	7.41 ± 2.24	7.55 ± 2.27	7.48 ± 1.91	7.49 ± 2.04	0.80
EQ-VAS (Mean ± SD)	80.88 ± 13.14	73.78 ± 17.81	78.7 ± 16.82	77.5 ± 16.88	0.14

**Figure 3:**
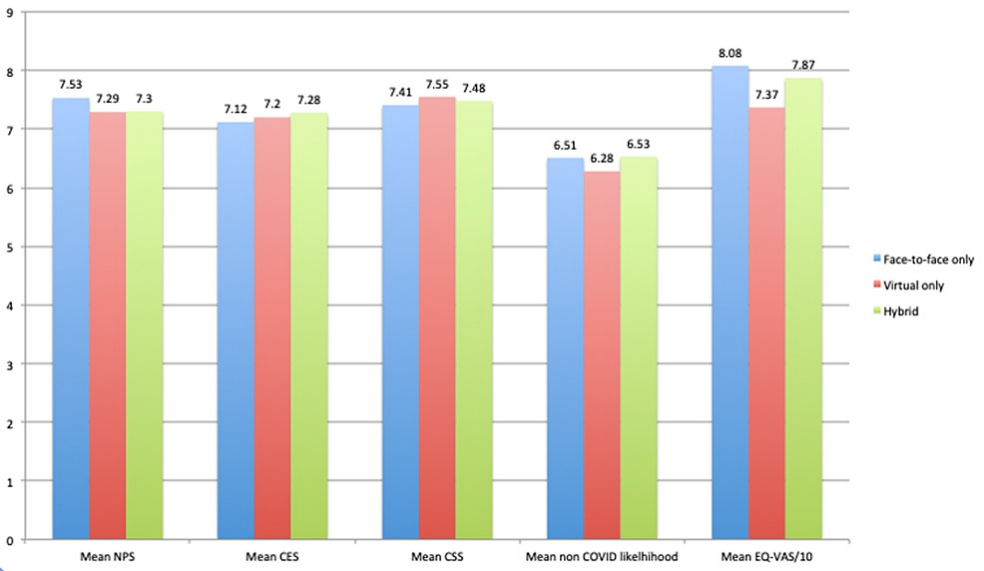
A comparison of the mean NPS, CES, CSS, non-COVID likelihood, and EQ-VAS scoring between patients with hybrid, only virtual, and only face-to-face consultations NPS: Net Promoter Score; CES: Customer Effort Score; CSS: Customer Satisfaction Score; EQ-VAS: EuroQol Visual Analogue Scale; COVID: coronavirus disease

Patients who had hybrid consultations tended to report better NPS, CES, and EQ-VAS scores than those who had virtual consultations only, although this difference did not reach statistical significance. Patients with face-to-face consultations had the highest mean NPS of 7.53 (Figure 3).

**Figure 4:**
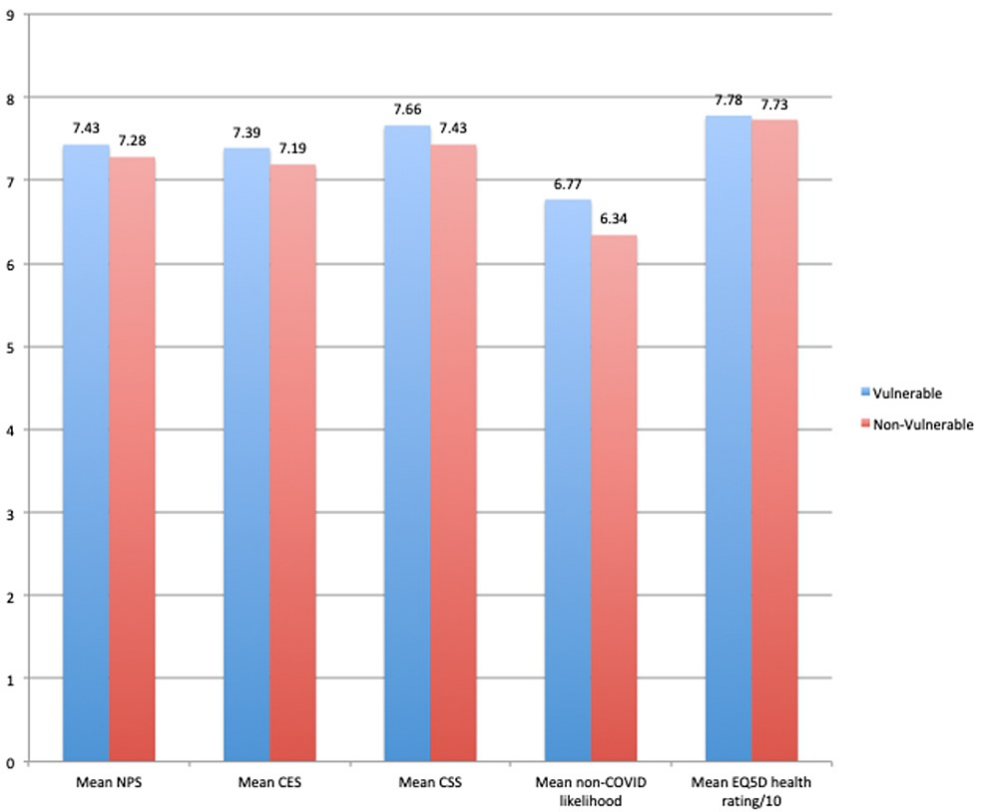
Comparison of NPS, CES, CSS, and EQ-VAS scores between vulnerable and non-vulnerable groups of patients in the HVFC NPS: Net Promoter Score; CES: Customer Effort Score; CSS: Customer Satisfaction Score; EQ-VAS: EuroQol Visual Analogue Scale; HVFC: hybrid virtual fracture clinic

Patients in the vulnerable group tended to report better overall mean scores than the non-vulnerable group including a higher mean EQ-VAS health score, but this difference did not reach statistical significance (p = 0.95) (Figure 4).

Patients who required surgery as part of their treatment reported worse overall mean scores than those who did not, although this difference did not reach statistical significance (Table 4). 

**Table 4:** Comparison of outcome measures depending on the type of management (surgical vs. non-surgical) and discharge status from HVFC HVFC: hybrid virtual fracture clinic

	Surgery?			Discharged?		
	Yes	No	p-value	Yes	No	p-value
Number of patients (%)	53 (30.99%)	118 (69.01%)		112 (65.5%)	59 (34.5%)	
Mean NPS ± SD	7.13 ± 1.89	7.41 ± 1.8	0.277	7.4 ± 1.81	7.17 ± 1.88	0.294
Mean CES ± SD	7.15 ± 1.91	7.28 ± 1.96	0.482	7.37 ± 2	7 ± 1.82	0.144
Mean CSS ± SD	7.23 ± 2.15	7.61 ± 1.99	0.272	7.62 ± 2.06	7.24 ± 2	0.137
Mean EQ-VAS ± SD	76.87 ± 19.96	77.79 ± 15.38	0.533	78.98 ± 16.02	74.69 ± 18.22	0.117

**Figure 5:**
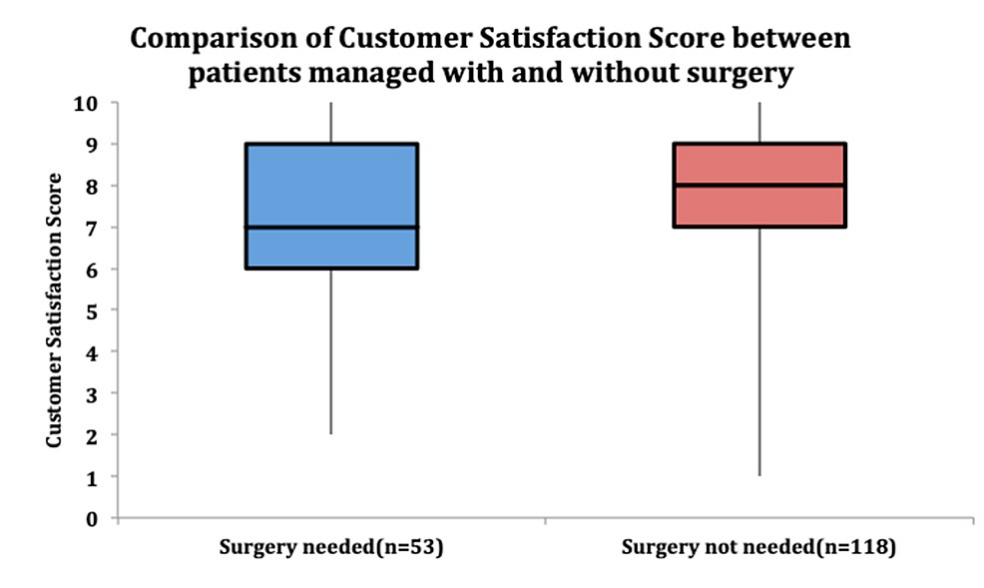
Box-Whisker plot depicting higher median customer satisfaction scores of patients managed without surgery

There was a significant difference in the pain component rating of the EQ-5D-5L, with surgical patients reporting higher pain levels compared to patients managed non-surgically (p = 0.01). Patients who did not require surgery tended to report increased customer satisfaction scores, as represented in the Box-Whisker plot (Figure 5).

Patients already discharged from the service reported higher overall mean scores including a higher EQ-VAS (Table 4). There was a significant difference between the pain component of EQ-5D-5L, with patients still under follow-up reporting higher pain levels (p < 0.001). Patients who were still under follow-up also reported significantly higher anxiety levels compared to those discharged (p = 0.002) on the EQ-5D-5L questionnaire.

## Discussion

The NHS is facing increasing pressures due to the COVID-19 pandemic, affecting the delivery of all services, including orthopaedic fracture clinics. The pandemic has highlighted the significance of remote consultations to reduce hospital visits and has led to newfound practices with a fresh interest in the efficiency and efficacy of virtual clinics across all specialties. The given circumstances resulted in the BOA recommending only unavoidable patient-initiated follow-ups in fracture clinics through new guidelines during the pandemic [4]. The need to protect the vulnerable group from COVID-19 exposure held particular importance while planning virtual clinics. In our hospital, this need, combined with the need to reduce overall patient footfall, resulted in the formulation of the hybrid virtual fracture clinic.

Previous studies have suggested high satisfaction ratios and acceptance of virtual fracture clinics among patients; however, all of these have focused solely on virtual consultations [7]. To the best of our knowledge, no previous study has evaluated the outcomes and patient satisfaction with a hybrid format of the clinic. The hybrid virtual fracture clinic (HVFC) format was bespoke to patient need - combining virtual and face-to-face consultations as appropriate for management.

Our results indicate that patient satisfaction and clinical outcomes with virtual and hybrid formats of consultations are similar to those with traditional face-to-face consultations. Also, patients with at least one face-to-face consultation in combination with virtual consultations, tend to have better satisfaction and outcome scores than those with only virtual consultations.

Patients vulnerable to COVID-19 have reported excellent outcomes across all measures in our study. Their superior satisfaction scores compared to non-vulnerable patients, highlight their recognition of the HVFC enabling them to isolate themselves at home while being provided effective outpatient care.

Our results also show that patients with orthopaedic conditions not requiring surgery may be most suited to this hybrid format, as such patients tend to report better satisfaction and clinical outcome measures compared to those requiring surgical management. The relatively lower satisfaction scores in surgical patients may partly be due to higher pain levels, as highlighted by the significantly higher rating of the pain component of EQ-5D-5L in patients requiring surgery.

The HVFC has the significant benefit of minimizing hospital visits of patients vulnerable to COVID-19 while continuing to provide clinical outcomes comparable to face-to-face consultations. Our results show that with this hybrid format, we were able to reduce footfall by 55.65%, with equivalent patient satisfaction and outcomes. The HVFC format could provide an efficient and economical model for conducting fracture clinics in the future, reducing the burden of face-to-face consultations. 

Another important benefit of the HVFC is reducing travel, especially in the current era of climate change, with a huge emphasis being on promoting sustainable healthcare. There is a drive towards devising strategies to make the NHS and surgical practice overall, more environment friendly [12]. The HVFC format, reducing travel to clinic appointments by over 50%, while providing efficient outpatient care, can certainly help promote this.

Technological advances have highly enabled televidimedicine [13] and virtual clinics have been studied in the past regarding their outreach to remote locations, and their economical benefit [14]. Virtual fracture clinics (VFCs) have been initiated in the United Kingdom previously with the intention to meet the British Orthopaedic Association Standards for Trauma (BOAST) guideline that requires a trauma patient to be reviewed at a fracture clinic within 72 hours of presenting to the emergency department, and have been found to be in compliance with this guideline [15,16]. We attribute our low compliance to this guideline, to the timing of our study at the commencement of lockdown, and re-organisation of clinic lists during this period, resulting in a delay in new appointments.

Potential cost reduction is another benefit of an HVFC model. The Glasgow Fracture Pathway concluded a cost saving of approximately 38% using virtual consultations [17]. Similarly, McKirdy and Imbuldeniya concluded that an amount of 67385.67 GBP was conserved within the first year of implementation of virtual clinics, and predicted to save approximately 129885.67 GBP every year after that [7]. Also, the incorporation of virtual consultations can increase the overall capacity of the fracture clinic. Logishetty and Subramanyam showed a rise of 8% in the total outpatient fracture appointments after the implementation of a virtual fracture clinic [18].

Rhind et al. did a literature review that indicated the advantages of merging VFCs and face-to-face consultations. In their review, 91-97% of patients were satisfied after VFC consultations. A decrease in non-attendance of almost 75% was noted. They also noted that VFCs do not impede the training and education of trainees [19].

As we included patients with varying orthopaedic conditions, we are unable to comment, from our results, on whether patients with certain conditions may fare better with the HVFC than others. However, previous studies on VFCs have shown their effectiveness for the management of a number of orthopaedic injuries not requiring surgical intervention. These include patients with simple radial head fractures, little finger metacarpal fractures, fifth metatarsal fractures, and buckle fractures in children [20,21]. Bhattacharya et al. found that patients with undisplaced clavicle fractures can be discharged after a single virtual consultation with good patient outcomes and satisfaction [22]. Similarly, the BOA COVID guidelines now encourage early definitive management of such injuries with virtual/patient-initiated follow-ups, in order to minimise subsequent visits to the fracture clinic [4].

Previous VFCs have been shown to have good outcomes in terms of patient satisfaction. In the study by Hawarden et al., the VFC was thought to be good or excellent by 94% of responders. Ninty-seven percent responded that they would be likely or extremely likely to suggest the VFC to family members or friends [23]. Jayram et al. showed satisfaction ranging from 87% to 95% in patients with radial head or neck fractures managed solely with virtual consultations [24].

## Conclusions

The HVFC format reduced patient footfall significantly (>50%) while providing effective and satisfactory outpatient care during the first COVID-19 wave. There was no difference in patient-reported outcomes between face-to-face consultations, hybrid, or virtual consultations thus showing that the HVFC is a safe and efficacious method for outpatient management of orthopaedic trauma, in line with BOA guidelines. Patients would recommend this service to others, they found it easy to use and reported a good experience. It enables patients to stay at home, which in turn eases pressure on public healthcare, which could, in turn, save lives in keeping with United Kingdom public health promotion. 
